# Correction: The computational nature of memory modification

**DOI:** 10.7554/eLife.28693

**Published:** 2017-05-22

**Authors:** Samuel J Gershman, Marie-H Monfils, Kenneth A Norman, Yael Niv

Gershman SJ, Monfils M-H, Norman KA, Niv Y. 2017. The computational nature of memory modification. *eLife* **6**:e23763. doi: 10.7554/eLife.23763.

Published 15, March 2017

A reader recently pointed out two errors in the originally published article.

In Figure 10, the schematic incorrectly described the experiment and simulation. The protein synthesis inhibitor was injected *after conditioning*, not at the time of retrieval. The schematic has been corrected. The error does not substantively change the scientific conclusions. The caption of Figure 10 has been corrected as follows:

“The amnestic affect of PSI administration after conditioning can be reversed by readministering the PSI at the time of test.”

Likewise, the text incorrectly stated that the protein synthesis inhibitor was injected at the time of retrieval. The text has now been corrected as follows:

“As in previous studies, the authors found that post-conditioning PSI administration disrupted the CR at test; the novel twist was that administering the PSI both immediately after conditioning and immediately before test eliminated the disruptive effect.”

The corrected Figure 10 is shown here:

**Figure 1:**
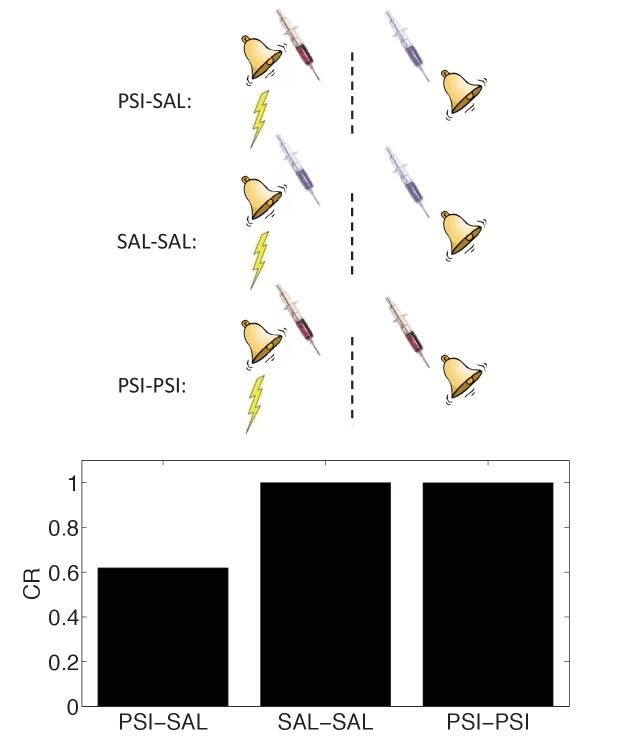

The originally published Figure 10 is also shown for reference:

**Figure 2:**
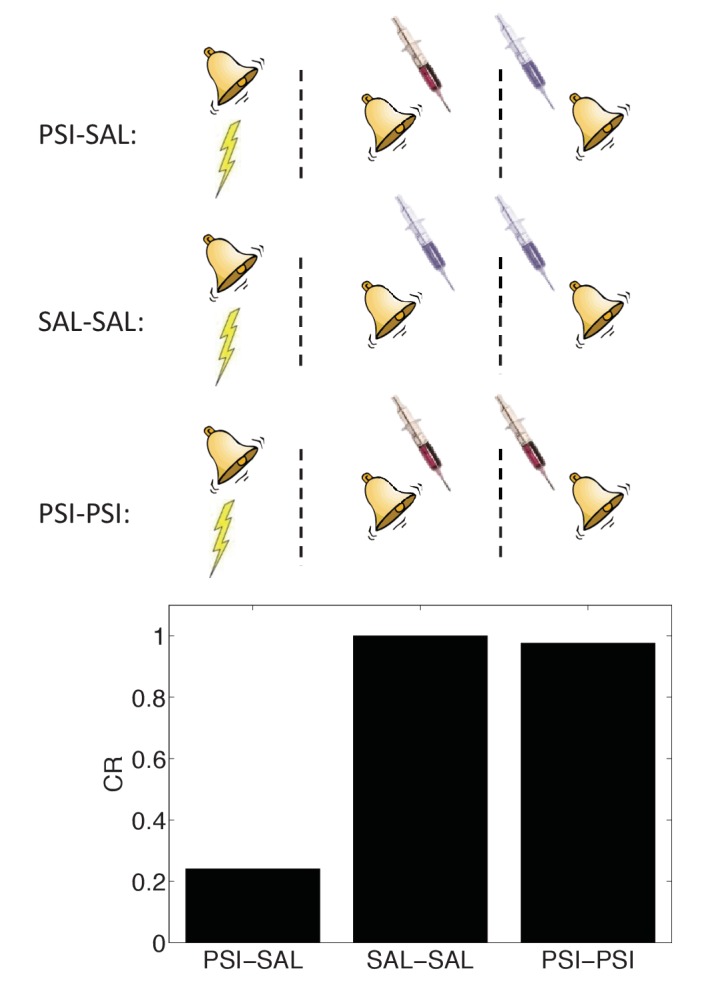

Owing to a production error the following sentence after equation 10 was erroneously omitted from the published version:

“where $\Phi (\cdot ;{\tilde{r}}_{t},\lambda )$ is the Gaussian cumulative distribution function with mean ${\tilde{r}}_{t}$ and variance λ. One way to understand Eq. 10 is that the animal’s conditioned response corresponds to its expectation that the US is greater than some threshold, θ. When $\lambda =\sigma_{r}^{2}$ (the US variance), Eq. 10 corresponds precisely to the posterior probability that the US exceeds θ:”

This sentence has now been added back.

The article has been corrected accordingly.

